# Assembled Reduced Graphene Oxide/Tungsten Diselenide/Pd Heterojunction with Matching Energy Bands for Quick Banana Ripeness Detection

**DOI:** 10.3390/foods11131879

**Published:** 2022-06-24

**Authors:** Xian Li, Chengcheng Xu, Xiaosong Du, Zhen Wang, Wenjun Huang, Jie Sun, Yang Wang, Zhemin Li

**Affiliations:** 1 Agricultural Information Institute, Chinese Academy of Agricultural Sciences, Beijing 100081, China; lixian@caas.cn (X.L.); zhenskar@163.com (Z.W.); 2 State Key Laboratory of Electronic Thin Films and Integrated Devices, School of Optoelectronic Science and Engineering, University of Electronic Science and Technology of China, Chengdu 610054, China; xcc_uestc@163.com (C.X.); xsdu@uestc.edu.cn (X.D.); 202022050434@std.edu.cn (W.H.); sjjjya@163.com (J.S.); 3 Graduate School of Chinese Academy of Agricultural Sciences, Beijing 100081, China

**Keywords:** fruit quality monitoring, room-temperature ethylene sensor, density functional theory, adsorption energy, band energy alignment

## Abstract

The monitoring of ethylene is of great importance to fruit and vegetable quality, yet routine techniques rely on manual and complex operation. Herein, a chemiresistive ethylene sensor based on reduced graphene oxide (rGO)/tungsten diselenide (WSe_2_)/Pd heterojunctions was designed for room-temperature (RT) ethylene detection. The sensor exhibited high sensitivity and quick p-type response/recovery (33/13 s) to 10–100 ppm ethylene at RT, and full reversibility and excellent selectivity to ethylene were also achieved. Such excellent ethylene sensing behaviors could be attributed to the synergistic effects of ethylene adsorption abilities derived from the negative adsorption energy and the promoted electron transfer across the WSe_2_/Pd and rGO/WSe_2_ interfaces through band energy alignment. Furthermore, its application feasibility to banana ripeness detection was verified by comparison with routine technique through simulation experiments. This work provides a feasible methodology toward designing and fabricating RT ethylene sensors, and may greatly push forward the development of modernized intelligent agriculture.

## 1. Introduction

Ethylene is an important plant hormone that regulates the physiological and biochemical changes in climacteric fruits and vegetables to control their maturity, freshness, softness, and deterioration [1,2]. The released ethylene can potentially express the flavor quality of fruits coupled with sugar content [3,4]. The accumulated ethylene molecules inside a fresh fruit package could also stimulate physiological activity and consequently, accelerate fruit deterioration, which limits their storage life and leads to product losses [5]. Moreover, the wounding and spoilage of fruits also induces the biosynthesis of ethylene [6]. According to a report from the Food and Agriculture Organization (FAO), 1.3 billion tons of food loss per year was reported, which represented 33% of total food production, among which fruits and vegetables held the highest loss rate (45%) [7]. Fruit abnormalities in early stages can be discovered promptly through ethylene monitoring and thus most of these losses could be avoided. Moreover, for the timely export of fresh climacteric fruits, the production as well as respiration of ethylene should also be of concern during long-supply chains [8]. Therefore, the continuous and accurate detection/monitoring of ethylene released from fruits is vital for managing and controlling the harvesting, storage, package, transportation, and selling process of climacteric fruits, which is much more prominent especially in today’s intelligent agriculture era.

So far, various techniques including chromatography [9], spectroscopy [10,11], electrochemical sensor [12], chemical sensor [13], and fluorescence probe [14] have been reported for ethylene detection, among which chemical sensor stands out, owing to its real-time response, high sensitivity, manpower operation, and low cost. Recently, many attempts have been made to develop high-performance chemiresistive ethylene sensors, in which the gas concentration is translated into a resistive electrical signal for detection. Bulk or nanostructured metal oxide semiconductors (MOSs, WO_3_ [15], SnO_2_ [16], and ZnO [17]) have been reported to show high sensitivity to ethylene. However, in metal oxide-based ethylene sensors, high temperature (typically 170–500 °C) or light irradiation is usually required to activate the reaction between adsorbed oxygen and ethylene, which results in high system complexity and high power consumption and thus greatly hinders its practical applications. Therefore, how to achieve high sensitivity to ethylene without additional activation energy is of great urgency and importance for practical agricultural applications due to its merits in reducing power consumption and system complexity. For MOSs, doping with noble metals such as Au, Pt, Pd [18,19,20], and transition metal halides (CuCl_2_, NiCl_2_ [21]) has been demonstrated to greatly lower the operating temperature. However, how to achieve excellent ethylene sensing performances at room temperature is still challenging.

The construction of a sensing material system for room-temperature ethylene detection should be considered from the following two aspects. Firstly, the gas sensing response arises from the physical adsorption of ethylene molecules onto the sensing films, and thus the adsorption capabilities of the sensing films should be optimized to achieve high sensitivity toward ethylene. It has been theoretically proven that the negative adsorption energy of the target analyte-sensing film system is beneficial for the adsorption of target analyte molecules, which also has been proven experimentally [22,23]. Secondly, when the target analyte molecules were adsorbed onto the sensing film, the electron transfer takes place between the target analyte and sensing film [24,25]. How to translate this electron transfer process into electrical resistance change greatly depends on the energy level alignment of the sensing material system, where suitable energy alignment promotes the electron transfer and thus results in a larger sensing response.

Following this regard, the ternary reduced graphene oxide (rGO)/tungsten diselenide (WSe_2_)/Pd heterojunctions were designed and fabricated toward room-temperature ethylene detection. The negative adsorption energy of the ternary heterojunctions provides enough active sites for ethylene molecules adsorption, and the electron transfer across the rGO/WSe_2_ and WSe_2_/Pd interfaces through band energy alignment greatly promotes the sensing response. Compared to the solely one or two components-based heterojunctions, the ternary heterojunction-based ethylene sensor exhibits higher sensitivity and quicker p-type response to the ppm level of ethylene at room temperature, and the sensitivity to 10 ppm of ethylene was 0.001%, with the response and recovery time being 33 and 13 s. Moreover, the sensor exhibits full reversibility and excellent selectivity at room temperature. Furthermore, the application feasibility of the sensor to fruit quality monitoring was verified by comparison with routine techniques through banana ripeness detection simulation experiments. This work provides a feasible methodology for designing and fabricating a sensing material system toward room-temperature ethylene detection, pushing forward the development of modernized intelligent agriculture.

## 2. Materials and Methods

### 2.1. Preparation

GO aqueous solution (0.5 mg/mL, Hangzhou Gaoxi Tech Co., Ltd., Hangzhou, China), WSe_2_ nanosheets dispersion (0.5 mg/mL, Nanjing MKNANO Tec Co., Ltd., Nanjing, China), and Pd nanoparticles (NPs, 99.9% metals basis, ≤1 μm, Shanghai Aladdin Biochemical Tech Co., Ltd., Shanghai, China) were prepared. Polyelectrolytes poly (diallyldimethylammonium chloride) (PDDA, 200,000–350,000, 20 wt. % aqueous solution, polycation), and poly (sodium-pstyrenesulfonate) (PSS, 70,000, polyanion) were purchased from Sigma-Aldrich. PDDA and PSS were diluted and dissolved in deionized (DI) water to 1 wt. % and 2 wt. %, respectively. Pd NPs were then dispersed in PSS solution with a concentration of 0.5 mg/mL. All reagents were directly used without further purification.

### 2.2. Sensor Fabrication

Ethylene sensitive films were deposited on SiO_2_/Si substrates pre-patterned with Ti/Au (20 nm/50 nm) interdigital electrodes (IDEs) by the layer-by-layer self-assembling method. The cleaned substrate was vertically immersed in PDDA solution for 15 min and dried with nitrogen, followed by immersing in DI water for removal of excessive PDDA molecules. Therefore, positive charges were fixed on the surface of substrates. Since the GO, WSe_2_, and PSS-Pd solutions were negatively charged, the positive charged substrate was then vertically immersed in negatively charged solution for 15 min for self-assembling of nanostructures on IDEs due to the electrostatic adsorption effects. Finally, the sensing films were annealed at 200 °C for 1 h to reduce GO. 

### 2.3. Instruments and Measurements 

The surface morphology and microstructure of sensing materials and films were observed by scanning emission microscopy (SEM, Zeiss Gemini, Oberkochen, Germany) and transmission electron microscope (TEM, FEI G2F20, Hillsboro, OR, USA). The chemical compositions and surface states of the samples were examined by X-ray photoelectron spectroscopy (XPS, Thermo Sacalab 250Xi, Waltham, MA, USA). The Raman spectra were recorded with He-Ne laser excitation at 532 nm using a Raman spectrometer (Renishaw inVia, Wotton-under-Edge, UK).

The ethylene-sensing properties of the sensors were measured at room temperature by a homemade dynamic test system (Figure S1) [26]. The ethylene standard gas (10–100 ppm in dry air), main interference gas (CO_2_, 30,000 ppm in dry air), and carrier gas dry air were supplied by Chengdu Xuyuan Chemical Co., Ltd., Chengdu, China. The concentration of tested ethylene and CO_2_ was controlled by the mass flow control (MFC300, Wuxi Aitoly Electronics Co., Ltd., Wuxi, China) with the dry air as carrier gas. The fabricated ethylene sensors were put into the test chamber and their resistances were recorded by a real-time multimeter resistance acquisition system (Keithley 2700, Cleveland, OH, USA). The sensing response of the sensor was defined as (R_g_ − R_0_)/R_0_, where R_g_ and R_0_ represent the steady-state resistance value of the sensor in the tested gas atmosphere and dry air, respectively. The response/recovery time was defined as the time required for 90% change of the resistance during the adsorption/desorption process.

### 2.4. Theoretical Calculation

DFT (Density Functional Theory) calculations were carried out using the Vienna Ab-initio Simulation Package (VASP) with the frozen-core all-electron projector-augment-wave method. The Perdew—Burke—Ernzerhof (PBE) of Generalized Gradient Approximation (GGA) was adopted to describe the exchange, correlation potential, and structure optimization. Van der Waals interactions were considered by the DFT-D2 method of Grimme. The plane wave basis set cut-off energy was set to 500 eV, and the Monkhorst-Pack k-point sampling was set to 2 × 2 × 1. The geometry optimizations were performed until the forces on each ion was reduced below 0.01 eV/Å.

The adsorption energy (E_ads_) was calculated as:

E_ads_ = E_adsorbent+gas_ – (E_adsorbent_ + E_gas_) (1)

where E_adsorbent_ was the energy of the sensitive materials, E_gas_ represented the energy of the adsorbed ethylene molecule, and E_adsorbent+gas_ was the total energy of the adsorbed system. Energetically, the negative adsorption energy values are desirable for the adsorption process.

### 2.5. Banana Ripeness Detection Experiments

The performance verification of the as-fabricated ethylene sensor was conducted by applications in banana ripeness detection. Yellowish green bananas were obtained from the local market and stored at room temperature (20 °C). During storage, the color of the samples changed with the increase of storage time, and the four typical color stages that are yellowish green, all yellow, yellow with brown speckles, and brown were chosen for detection of released ethylene. The bananas at the target sampling stage were put into a sealed quartz glass container. The containers were sealed for a period of time for collection of ethylene, and then the sensor was placed into the container as well to record its in-time resistance values for the detection of released ethylene. After sensing for 100 s, the sensor was taken out from the container, and placed in ambient air to recover to the initial resistance state. The above steps were repeated four times to obtain the sensing response to bananas at different ripeness stages. At the same time, the gas in the container was also extracted by a gas syringe (25 μL, VICI Precision Sampling, Inc., Baton Rouge, LA, USA) for gas analysis by gas chromatography-mass spectrometry (Agilent GC-MS 7890B-5977A, Santa Clara, CA, USA). The parameters of GC-MS were set as: 250 °C inlet temperature, 40 °C column temperature for 3 min, 40 °C /min speed to 100 °C, 1 mL/min flow rate, splitless, 230 °C ion source, and 150 °C MS quadrupole temperature. Identification of ethylene was confirmed by comparing the collected mass spectra with the spectra in the National Institute for Standards and Technology (NIST 14) data bank. The relative content of the ethylene was determined using the area normalization method. Three sampling analysis was one replicate and the average results were used.

## 3. Results

### 3.1. Theoretical Design of the Ethylene Sensitive Material System

**Figure 1:**
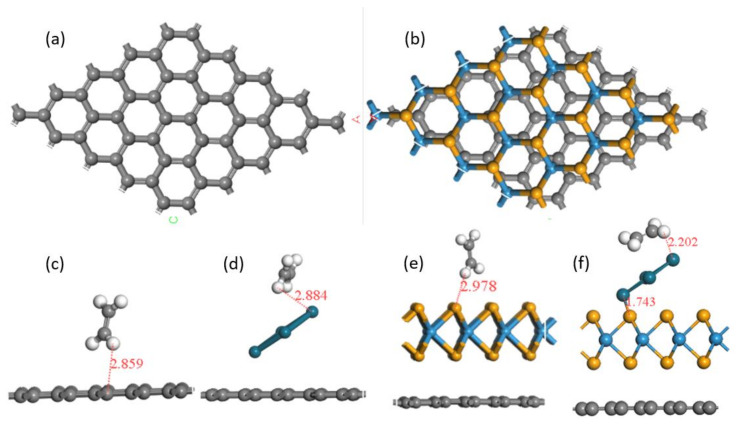
Optimized geometric models of (**a**) graphene and (**b**) graphene/WSe_2_, and adsorption structures of ethylene molecules on (**c**) graphene, (**d**) graphene/Pd, (**e**) graphene/WSe_2_, and (**f**) graphene/WSe_2_/Pd.

A model of graphene consisting of 55 atoms was constructed. The length of the C-C bond in basal plane was 1.42540 Å, which was close to the C-C bond length of the graphite planar structure [27]. The geometric structure of the 16 monolayer WSe_2_ was also discussed [28,29] and the length of the W-Se bond varied from 2.51006 to 2.51244 Å. The 4 Pd atoms with each volume of 62.01 Å^3^ were adopted. Lattice constants of graphene and WSe_2_ were calculated as 12.34 and 13.26, respectively. After compounding, the lattice constant of graphene/WSe_2_ was 12.64, indicating graphene was stretched while WSe_2_ was compressed. The length between ethylene and graphene, WSe_2_, and Pd, were 2.86 Å, 2.88 Å, and 2.98 Å, respectively, forming no chemical bonds and declaring van der Waals adsorption, as shown in Figure 1. While in ternary structure, there was a chemical bond (1.74 Å) linked between Pd and WSe_2_, and the distance between ethylene and WSe_2_ was shortened to 2.2 Å, indicating that the adsorption had been changed.

Energies of the above structures and the ethylene adsorbed on the structures were obtained (Table 1). After assemble with WSe_2_, the energy of graphene almost doubled. While after metal Pd modification, the energy of the graphene became slightly more powerful. Adsorption energies were calculated and are shown in Table 2. The adsorption of graphene to ethylene was only −0.14; a negative value indicated the adsorption occurred energetically and a weak value declared the van der Waals adsorption. Compounded Pd or WSe_2_ helped to enhance the adsorption capacity of graphene to ethylene. It was noticed that capacity drastically increased to 5.5 times through ternary compounding.

### 3.2. Fabrication and Characterization of Ethylene Sensitive Films

**Figure 2:**
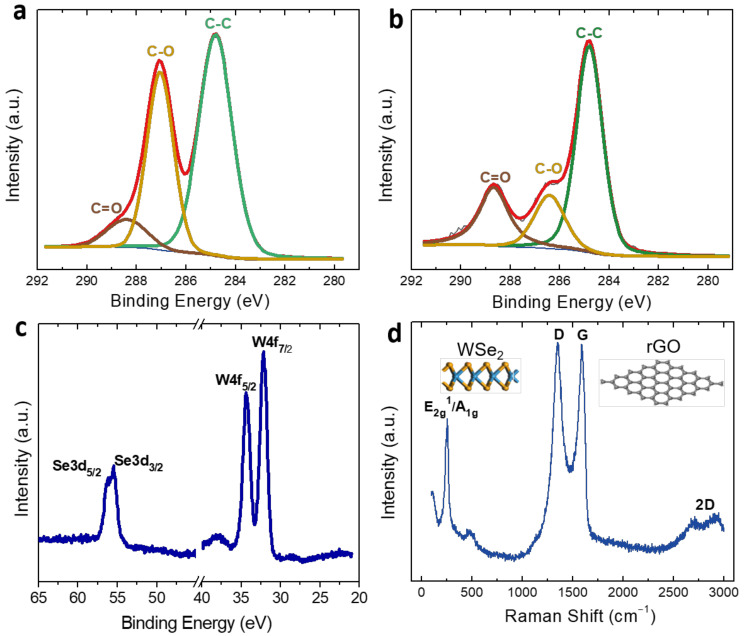
XPS C 1s spectrum of (**a**) GO and (**b**) rGO. (**c**) XPS spectra of W 4f and Se 3d of WSe_2_. (**d**) Raman spectrum of rGO/WSe_2_ sensitive films.

Four sensitive material systems were fabricated including rGO, rGO/WSe_2_, rGO/Pd, and rGO/WSe_2_/Pd to achieve the most promising candidates for room-temperature ethylene sensing. Firstly, the self-assembled films were characterized by XPS to investigate their chemical composition and surface states. Figure 2a,b shows the C 1s spectra of GO and rGO films, respectively. For GO films, C-C, C-O, and C=O are the three main types of carbon bonds, corresponding to the characteristic peaks at 284.8 eV, 287.0 eV, and 288.4 eV. After annealing, the peak of the C-O bond decreases greatly and the C-C bond becomes the main peak, indicating that most of the oxygen-containing functional groups in GO are removed and thus GO is reduced. After deposition of WSe_2_ nanosheets, the chemical composition of the rGO/WSe_2_ sensitive films was examined by XPS as well, shown in Figure 2c. Two obvious peaks appeared at 34.2 and 32.1 eV and were attributed to W4f_7/2_ and W4f_5/2_ of W^4+^, respectively, while the peaks at 54.2 eV and 55 eV correspond to the Se3d_3/2_ and Se3d_5/2_ of Se^2-^. Then, the Raman spectrum was also recorded to exhibit the structural properties of the rGO/WSe_2_ sensitive films, as displayed in Figure 2d. The characteristic peak of WSe_2_ at 251 cm^−1^ is clearly observed, which could be attributed to the overlapped peaks of the interlayer mode A_1g_ (253 cm^−1^) and in-plane vibration mode E^1^_2g_ (250 cm^−1^). The peaks at 1349 cm^−1^ and 1590 cm^−1^ correspond to the D and G band of rGO, respectively, and its intensity ratio is almost 1.0, indicating the reduction of GO into rGO with some defects. Moreover, the broad 2D band at 2470–3000 cm^−1^ shows that the fabricated rGO film is composed of some layers of the rGO sheets.

**Figure 3:**
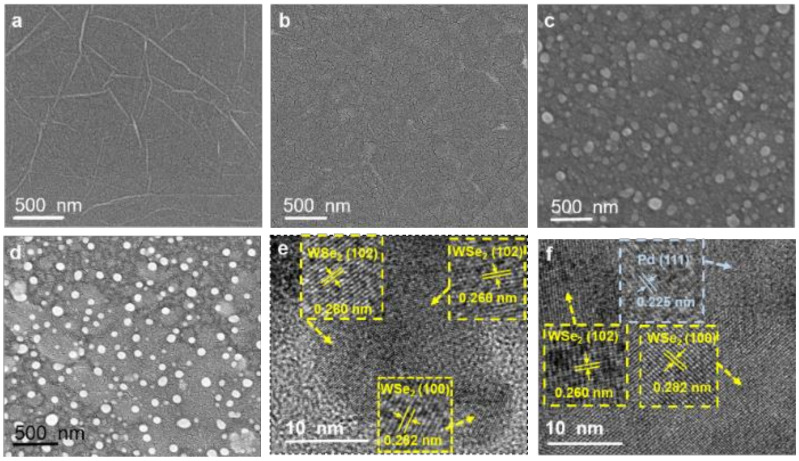
SEM images of the self-assembled (**a**) rGO, (**b**) rGO/WSe_2_, (**c**) rGO/Pd, and (**d**) rGO/WSe_2_/Pd, high-resolution TEM images of (**e**) rGO/WSe_2_ and (**f**) rGO/WSe_2_/Pd.

The surface morphology of rGO and its heterojunction films were observed through SEM. The pure rGO films show uniform morphology with a few wrinkles (Figure 3a). After deposition of lamellar WSe_2_ nanosheets, the rGO/WSe_2_ composite films remain uniform with excellent interlayer coupling between rGO and WSe_2_ nanosheets (Figure 3b,c). As shown in Figure S2, the EDS element mapping images of W, Se, C, and O confirm that these four elements are homogeneously present in the whole film region, which further proves formation of rGO/WSe_2_ heterojunction. Pd NPs are evenly distributed on rGO and rGO/WSe_2_ films (Figure 3c,d), which was beneficial for the sensing enhancement effects of Pd NPs. The corresponding high-resolution TEM (HRTEM) images exhibit a specific lattice spacing of the (102) facet for WSe_2_, (100) facet for WSe_2_, and (111) facet for Pd, which is measured to be 0.260 nm, 0.282 nm, and 0.225 nm, respectively, as shown in Figure 3d,e.

### 3.3. Ethylene-Sensing Characteristics

**Figure 4:**
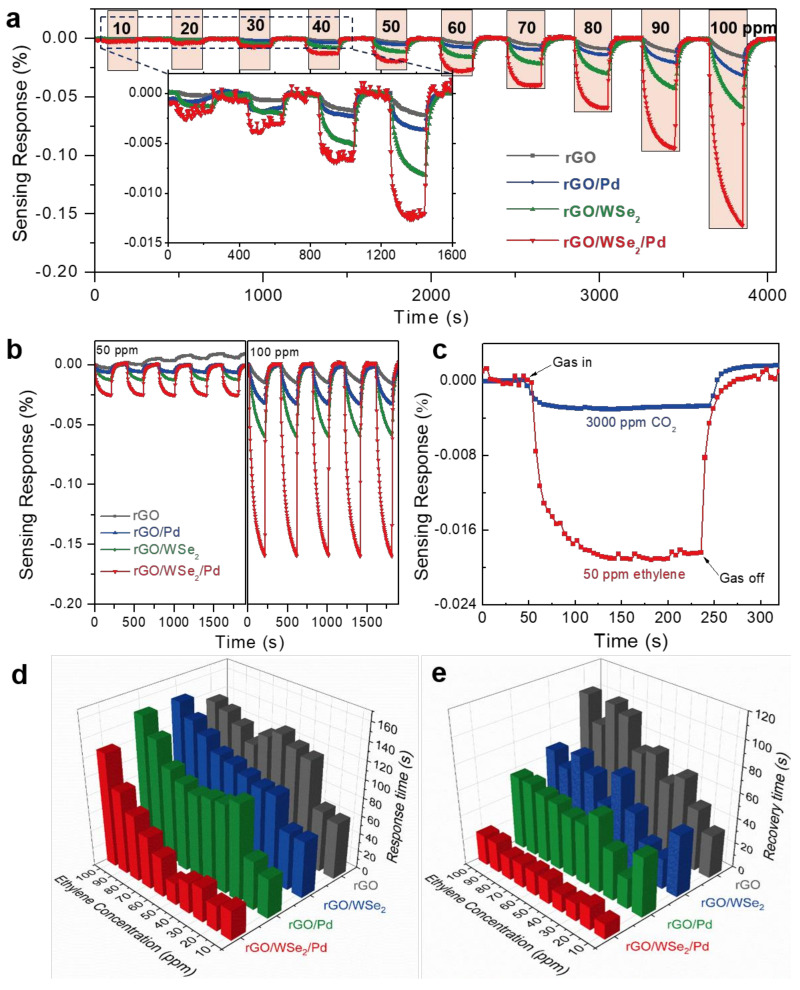
(**a**) Real-time sensing response of rGO-based self-assembled sensitive film to 10–100 ppm ethylene at room temperature, (**b**) repeatability, and (**c**) selectivity properties of self-assembled rGO/WSe_2_/Pd sensitive film when exposed to ethylene. (**d**) Response time and (**e**) recovery time of the ethylene sensor based on rGO/WSe_2_/Pd sensitive films.

Figure 4a shows the real-time sensing response versus time curves of four sensitive films (rGO, rGO/WSe_2_, rGO/Pd, and rGO/WSe_2_/Pd) when exposed to 10–100 ppm ethylene at room temperature. When exposed to ethylene, all resistance values decline rapidly and then approximately reach the saturation states after almost the same periods of time. After ethylene was purged by dry air, the resistance values gradually recovered to their initial states, which indicates the p-type semiconducting behaviors of all fabricated sensing films. The sensing response of all sensors increases with the increase of ethylene concentration ranging from 10 to 100 ppm, and it could be clearly seen that both of the rGO/WSe_2_ heterostructures and Pd NPs could greatly promote the ethylene sensing characteristics. Among them, pure rGO film shows the lowest sensing response, and the response to 100 ppm ethylene is nearly twice and four times larger after assembling with Pd and WSe_2_, respectively, and particularly, ten times for rGO/WSe_2_/Pd heterojunctions, compared with pure rGO based sensors. These results are consistent with the theoretical analysis results shown above, which further demonstrates the enhancement effects of rGO/WSe_2_ heterostructures and Pd NPs for ethylene sensing.

The repeatability of all kinds of sensitive films to 50 and 100 ppm ethylene was measured and shown in Figure 4b. It could be clearly seen that except for the case of rGO films in 50 ppm ethylene, almost similar response curves including response value, response/recovery time, and also stable baselines are obtained in five successive cycles, indicating the excellent repeatability properties of sensitive films. Moreover, to further prove its practical application in the agricultural environment, the selectivity properties of rGO/WSe_2_/Pd composite films were investigated. The typical interfering gas when used for fruit ripeness detection is CO_2_, which is produced by the respiration of fruits. The sensing response of the composite sensor to 50 ppm ethylene is significantly larger than that of 3000 ppm CO_2_ (Figure 4c), verifying the excellent selectivity of the rGO/WSe_2_/Pd sensitive films in fruit ripeness detection application scenarios. In addition, the response and recovery time (90% change in sensor resistance) were extracted and shown in Figure 4d,e. Among four kinds of sensitive films, the rGO/WSe_2_/Pd sensitive film exhibits the shortest response time (33 s) and recovery time (13 s) to 10 ppm ethylene. It could be attributed to the 2D structures of the rGO and WSe_2_ nanosheets, Se vacancies existing in the surface of WSe_2_ films, and also high activity of Pd NPs.

### 3.4. Banana Ripeness Detection Applications

**Figure 5:**
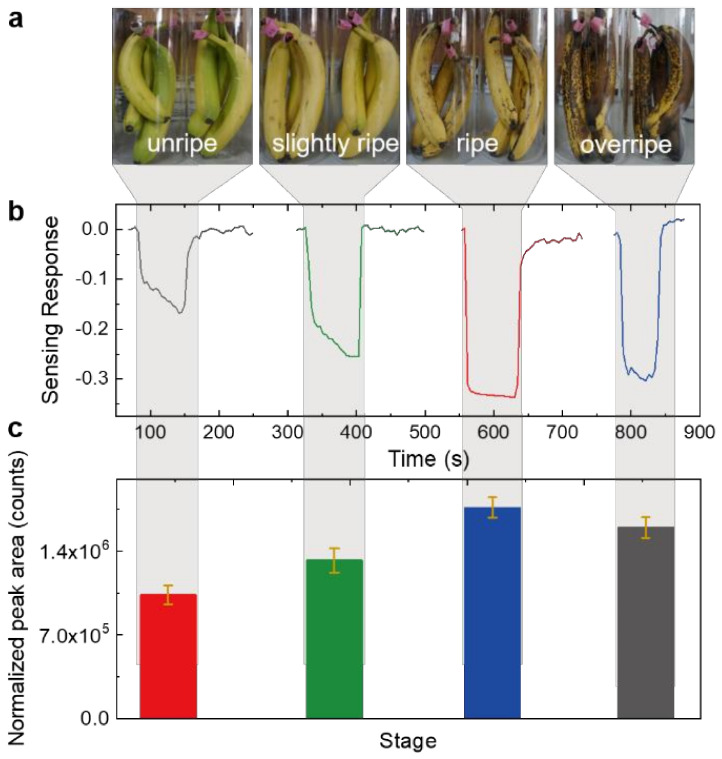
(**a**) Photos of banana samples at different storage stages: yellowish green (1st stage, unripe), all yellow (2nd stage, slightly ripe), all yellow with brown speckles (3rd stage, ripe), and dark brown (4th stage, overripe). (**b**) The real-time resistance versus time curves of the rGO/WSe_2_/Pd heterojunction-based chemiresistive sensor when placed into the sealed container with bananas at different ripeness stages. (**c**) The normalized peak area of released ethylene from banana samples at different ripeness stages by GC-MS technology.

The fruit ripeness detection experiments were then designed and conducted in the laboratory to verify the application feasibility of our as-fabricated ethylene sensors by comparison with traditional GC-MS technology. In our experiments, the bananas, a typical climacteric fruit, were adopted, and it had been demonstrated that ethylene was released during the ripeness process of bananas. The level of banana ripeness can be commonly divided into four stages: unripe, slightly ripe, ripe, and overripe [30], accompanying the gradual change of banana color and released ethylene concentration. The unripe bananas exhibit solid light green color with some light greenish-yellow, and show no significant aroma. Mostly yellow with very faint green at tips and along edges is defined as slightly ripe, which usually exhibits some faint banana aroma. After that, bananas step into the ripe stage, and the color become solid yellow with no green, but sporadic small brown spots. At this stage, the released aroma is the strongest. Afterwards, the bananas enter into the overripe stage with the color of dark brown and even black, during which the released aroma gradually drops into a relatively low level. According to the characteristics of the above four ripeness stages, the color changes of the obtained banana samples could be clearly observed (Figure 5a), and the bananas at different ripeness stages were placed into different contained. The containers were sealed for collection of ethylene, and after about 20 min, the sensors were placed into the containers for ethylene sensing toward banana ripeness detection. The sensing response to bananas at different ripeness stages is shown in Figure 5b, from which it could be clearly seen that the ethylene concentration increases gradually until the bananas reached the ripe stage. The sensing response is calculated to be 16.8%, 25.4%, 33.8%, and 30.2%, corresponding to the unripe, slightly ripe, ripe, and overripe bananas, respectively. The normalized peak area of the released ethylene measured by GC-MS exhibits the same changing trend with the banana ripeness stage i.e., the ethylene concentration increases until the bananas are ripe, and further declines when they become overripe, as shown in Figure 5c.

## 4. Discussion

According to the calculated adsorption energies, all the negative values manifested the advantage of room temperature operation of the fabricated ternary sensitive materials system with energy-level alignment, which may be caused by the formed chemical bond, further changing the energy band structure. The calculated adsorption energies were consistent with the sensing response of each adsorbed system to ethylene. Previous studies show that rGO is p-type materials. When ethylene molecules interact with p-type rGO sensitive films, electrons will transfer from rGO to ethylene, owing to higher Fermi levels of rGO than ethylene, resulting in a decrease of electron density of the rGO films and thus a negative sensing response to ethylene gas. When composited with Pd NPs, the sensing response to ethylene gas becomes more negative in rGO/Pd composites, which is inconsistent with the positive sensing response arising from the catalytic effects of Pd NPs toward ethylene molecules and thus excludes the role of catalytic properties on sensing response enhancements. This ethylene sensing enhancement could be attributed to the local doping effects of Pd NPs on rGO when the device is exposed to ethylene [31]. The large work function difference between high work function Pd NPs and rGO results in the local hole-doping of the rGO at the Pd NPs/rGO interfaces and thus lowers the Fermi energy levels of the rGO/Pd composites. Combined with the higher adsorption energy, both contributed to more electrons transferred from rGO/Pd to ethylene, and leads to an enhanced negative sensing response to ethylene.

In the case of rGO/WSe_2_ bilayer heterojunctions, the device resistance is dominated by conductive rGO films due to the bilayer device structure deposited on IDEs and much higher electrical conductivity of rGO than WSe_2_. When rGO and WSe_2_ are brought into contact, it is expected that electrons would pass from rGO to WSe_2_ until the equilibrium of the Fermi level is achieved. As a result, a Schottky-type junction is formed across the rGO/WSe_2_ interface with a downward band bending and a hole depletion region in WSe_2_ near the surface [32,33]. When the rGO/WSe_2_ heterojunction is exposed to ethylene, the electron transfer from WSe_2_ to the adsorbed ethylene molecules would lead to an increase of hole concentration in WSe_2_. The increased hole concentration in WSe_2_ causes a larger Fermi level difference between rGO and WSe_2_, leading to more holes transferred from WSe_2_ to rGO, and thus significantly increased sensitivity to ethylene. Furthermore, when Pd NPs are introduced to the rGO/WSe_2_ bilayer film, the Schottky junction effects are further enhanced by the hole doping effects in WSe_2_ induced by Pd NPs [34]. Combined with more negative adsorption energy of the rGO/WSe_2_/Pd composites, more holes will transfer from WSe_2_ to rGO, resulting in a further increased sensitivity to ethylene. Moreover, the unique structures of 2D materials and the self-assembled sensitive films could increase the adsorption sites for gas molecules, accelerate the charge transfer between rGO and WSe_2_ nanosheets, and promote the synergistic effects between them.

For climacteric fruit such as the banana, when it changes from ripe to overripe, the released ethylene concentration decreases. This indicates a direct relationship between released ethylene concentration and the banana ripeness stage, which still needs further quantitative research. Moreover, the released aroma of climacteric fruits has a positive correlation with the released inner ethylene during the storage process [3]. Therefore, intelligent ethylene sensor techniques cannot only reflect the ripeness information of climacteric fruits, but also can be potentially adopted to evaluate their flavor quality. The consistent results from our fabricated ethylene sensor and GC-MS confirm the reliability and feasibility of our proposed ethylene sensor methodology toward banana ripeness detection. Furthermore, the proposed ethylene sensor exhibits distinct advantages of low costs, fast and accurate detection, simple operation, and in situ monitoring. The ethylene sensors can also further be developed into electronic noses and integrated into picking robots, grading equipment, packaged boxes, and shelves, which could push forward the development of intelligent agriculture.

## 5. Conclusions

In conclusion, a room-temperature ethylene sensor based on a ternary rGO/WSe_2_/Pd heterojunction and its banana ripeness detection applications were demonstrated. The ethylene sensor shows excellent figures of merit including high sensitivity, rapid response/recovery, full repeatability, and high selectivity, making it promising for practical banana ripeness detection applications. Such excellent room-temperature ethylene sensing behaviors could be attributed to the following two aspects: (1) The negative adsorption energy of the ternary heterojunctions provides enough active sites for ethylene molecules adsorption, and (2) The electron transfer across the rGO/WSe_2_ and WSe_2_/Pd interfaces is greatly promoted through band energy alignment. Furthermore, its reliability and feasibility toward fruit quality monitoring applications were confirmed and validated through banana ripeness detection simulation experiments by comparison with traditional GC-MS technology. This work provides a feasible methodology for construction of a room-temperature ethylene sensing material system, and also may innovatively push forward the applications of sensor technology in the intelligent agriculture field to acquire the ripeness and quality information of climacteric fruit quickly and in-time.

## Figures and Tables

**Table 1:** The calculated single point energies of the adsorbed systems.

Structure	Energy (eV)
Ethylene	−31.75
Graphene	−458.56
Graphene/WSe_2_	−817.16
Graphene/Pd	−471.35
Graphene/WSe_2_/Pd	−805.71
Ethylene adsorbed on graphene surface	−490.45
Ethylene adsorbed on graphene/Pd surface	−503.27
Ethylene adsorbed on graphene/WSe_2_ surface	−849.10
Ethylene adsorbed on graphene/WSe_2_/Pd surface	−838.24

**Table 2:** Adsorption energies of the ethylene adsorption system.

Adsorbed System	Adsorption Energy (eV)
Ethylene-graphene	−0.14
Ethylene-graphene/Pd	−0.17
Ethylene-graphene/WSe_2_	−0.19
Ethylene-graphene/WSe_2_/Pd	−0.78

## Data Availability

Data are available from the authors.

## Supplementary Materials

The following supporting information can be downloaded at: https://www.mdpi.com/article/10.3390/foods11131879/s1, Figure S1: Schematic diagram of the gas sensing setup; Figure S2: EDS mapping results of the rGO/WSe_2_ heterojunction films.
