# Supplementary material for: Assembled Reduced Graphene Oxide/Tungsten Diselenide/Pd Heterojunction with Matching Energy Bands for Quick Banana Ripeness Detection
Source: Foods. 2022 Jun 24;11(13):1879. doi: 10.3390/foods11131879 (PMC9265317; doi:10.3390/foods11131879)
Supplement: Supplementary file 1 [file foods-11-01879-s001.zip › foods-1757263-supplementary.pdf]

Assembled Reduced Graphene Oxide/Tungsten Diselenide/Pd Heterojunction with Matching Energy Bands for Quick Banana Ripeness Detection

Xian Li [1], Chengcheng Xu [2], Xiaosong Du [2], Zhen Wang [1], Wenjun Huang [2], Jie Sun [2], Yang Wang [2,*] and Zhemin Li [1,3,*]

[1]Agricultural Information Institute, Chinese Academy of Agricultural Sciences, Beijing, 100081, PR China; lixian@caas.cn(X.L.); zhenskar@163.com(Z.W.)
[2] State Key Laboratory of Electronic Thin Films and Integrated Devices, School of Optoelectronic Science and Engineering, University of Electronic Science and Technology of China, Chengdu, 610054, PR China; xcc_uestc@163.com(C.X.); xsdu@uestc.edu.cn(X.D.); 202022050434@std.edu.cn(W.H.); sjjjya@163.com (J.S.); landlord@uestc.edu.cn (Y.W.)
[3]Graduate School of Chinese Academy of Agricultural Sciences, Beijing, 100081, PR China; lizhemin@caas.cn
*Correspondence: landlord@uestc.edu.cn (Y.W.); lizhemin@caas.cn (Z.L.)

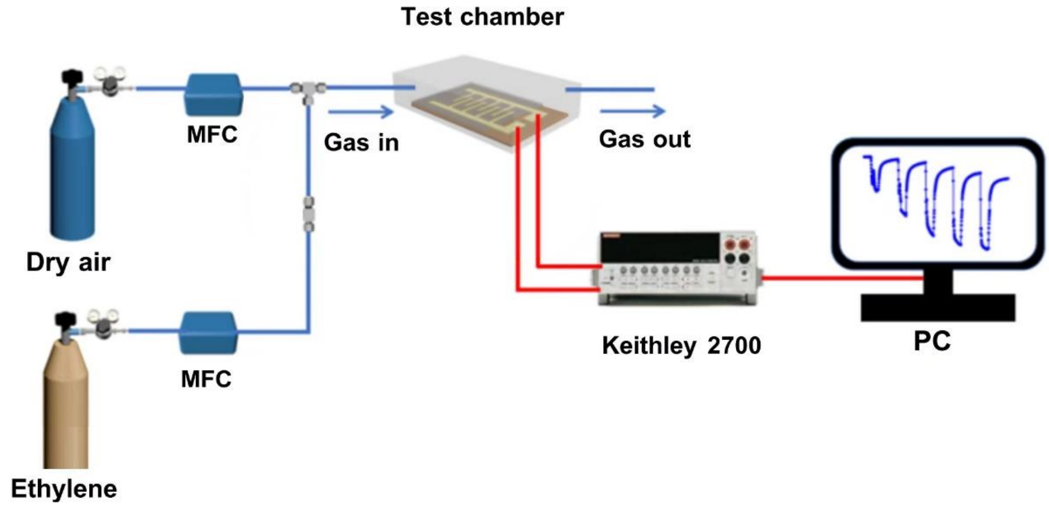

Figure S1. Schematic diagram of the gas sensing setup

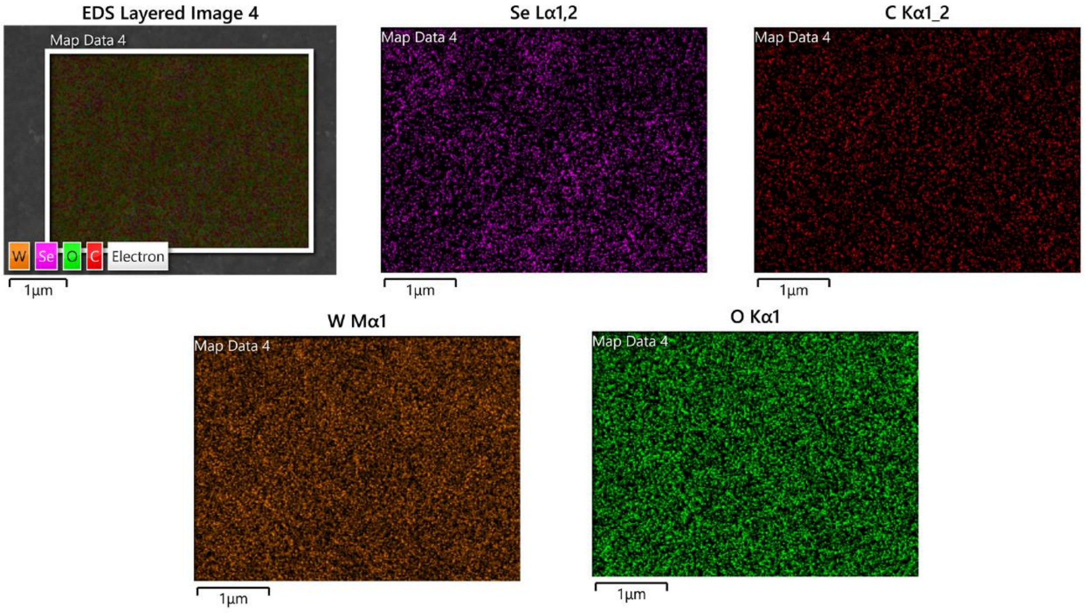

Figure S2. EDS mapping results of the rGO/WSe_2 heterojunction films.
